# Estradiol Protects against Noise-Induced Hearing Loss and Modulates Auditory Physiology in Female Mice

**DOI:** 10.3390/ijms222212208

**Published:** 2021-11-11

**Authors:** Benjamin Shuster, Ryan Casserly, Erika Lipford, Rafal Olszewski, Béatrice Milon, Shaun Viechweg, Kanisa Davidson, Jennifer Enoch, Mark McMurray, Mark A. Rutherford, Kevin K. Ohlemiller, Michael Hoa, Didier A. Depireux, Jessica A. Mong, Ronna Hertzano

**Affiliations:** 1 Department of Otorhinolaryngology—Head and Neck Surgery, University of Maryland School of Medicine, Baltimore, MD 21201, USA; benjamin.shuster@som.umaryland.edu (B.S.); ryan.casserly@gmail.com (R.C.); elipford@som.umaryland.edu (E.L.); bmilon@som.umaryland.edu (B.M.); mm2726@princeton.edu (M.M.); 2 Auditory Development and Restoration Program, National Institute on Deafness and Other Communication Disorders, NIH, Bethesda, MD 20892, USA; rafal.olszewski@nih.gov (R.O.); michael.hoa@nih.gov (M.H.); 3 Department of Pharmacology, University of Maryland School of Medicine, Baltimore, MD 21201, USA; stump86@gmail.com (S.V.); kdavidson@som.umaryland.edu (K.D.); jense674@gmail.com (J.E.); jmong@som.umaryland.edu (J.A.M.); 4 Department of Otolaryngology, Washington University School of Medicine, St. Louis, MO 63110, USA; rutherfordmark@wustl.edu (M.A.R.); kohlemiller@wustl.edu (K.K.O.); 5 Otolith Labs, Washington, DC 20036, USA; depireux@gmail.com; 6 Department of Anatomy and Neurobiology, University of Maryland School of Medicine, Baltimore, MD 21201, USA; 7 Institute for Genome Sciences, University of Maryland School of Medicine, Baltimore, MD 21201, USA

**Keywords:** noise-induced hearing loss, mouse model, estrogen, sex-differences, cochlear synaptopathy, inner ear, auditory physiology

## Abstract

Recent studies have identified sex-differences in auditory physiology and in the susceptibility to noise-induced hearing loss (NIHL). We hypothesize that 17β-estradiol (E_2_), a known modulator of auditory physiology, may underpin sex-differences in the response to noise trauma. Here, we gonadectomized B6CBAF1/J mice and used a combination of electrophysiological and histological techniques to study the effects of estrogen replacement on peripheral auditory physiology in the absence of noise exposure and on protection from NIHL. Functional analysis of auditory physiology in gonadectomized female mice revealed that E_2_-treatment modulated the peripheral response to sound in the absence of changes to the endocochlear potential compared to vehicle-treatment. E_2_-replacement in gonadectomized female mice protected against hearing loss following permanent threshold shift (PTS)- and temporary threshold shift (TTS)-inducing noise exposures. Histological analysis of the cochlear tissue revealed that E_2_-replacement mitigated outer hair cell loss and cochlear synaptopathy following noise exposure compared to vehicle-treatment. Lastly, using fluorescent in situ hybridization, we demonstrate co-localization of estrogen receptor-2 with type-1C, high threshold spiral ganglion neurons, suggesting that the observed protection from cochlear synaptopathy may occur through E_2_-mediated preservation of these neurons. Taken together, these data indicate the estrogen signaling pathways may be harnessed for the prevention and treatment of NIHL.

## 1. Introduction

Disabling hearing loss afflicts nearly half a billion people worldwide. Noise-induced hearing loss (NIHL)—a form of acquired hearing loss—comprises a significant portion of this global burden, and its incidence is expected to increase [1,2]. Despite its widespread prevalence, there are currently no FDA-approved therapeutics for the prevention or treatment of NIHL, leaving amplification (hearing aids) and cochlear implantation as the sole treatment options [3,4].

The severity of NIHL presents as a continuum. Small increases in the intensity or duration of a noise exposure result in the progression from a less severe to a more severe subtype [5]. The least severe subtype of NIHL—referred to as a temporary threshold shift (TTS)—results in a temporary elevation of auditory thresholds without loss of, or permanent damage to, the mechanosensory cells of the cochlea—inner hair cells (IHCs) and outer hair cells (OHCs)—or their associated auditory nerve fibers (ANFs) arising from the spiral ganglion neurons (SGNs) [6]. A more severe subtype of NIHL called cochlear synaptopathy also produces a TTS but causes uncoupling and/or dysfunction of the synaptic connections between IHCs and high-threshold, low-spontaneous rate SGNs, which appear to correspond to type-1C SGNs [7,8,9,10]. In humans, cochlear synaptopathy is believed to underpin ‘hidden hearing loss’ (HHL), a functional impairment that includes difficulty understanding speech in noise [9,11]. Finally, the most severe subtype of NIHL results in a permanent elevation of auditory thresholds—or permanent threshold shift (PTS)—with attendant damage to, or loss of, IHCs and OHCs. Following octave-band noise exposures, this loss often occurs half an octave above the noise band and in the more vulnerable basal portions of the cochlea, where basal OHCs display an intrinsic vulnerability to damage caused by free radicals compared to more apical OHCs [6,12,13,14]

While NIHL afflicts both males and females, recent evidence suggests that females display increased resilience to NIHL compared to males, and that this resilience may be mediated by estrogen signaling [15,16,17,18,19,20,21]. Recent findings from our laboratory demonstrate that gonadally intact female mice are relatively protected from a PTS-inducing noise exposure in comparison to gonadally intact males [16]. Furthermore, estrogen receptor-β (estrogen receptor 2 or ESR2) knockout (KO) mice of both sexes are more susceptible to TTS-inducing noise exposures than their wildtype littermate controls, while female ESR2 KO mice display early onset age-related hearing loss (ARHL) [22,23]. Both findings suggest that this protective effect may be, at least in part, mediated via ESR2-signaling.

The apparent protective effects of the estrogen signaling pathways offer a compelling avenue of investigation into their therapeutic potential. In the present study, we utilize a surgical gonadectomy model to characterize the effects of estrogen removal and replacement on peripheral auditory physiology in the absence of noise exposure. The surgical gonadectomy model eliminates circulating gonadal steroids including endogenous E_2_ in mice of both sexes, as E_2_ is generated in males by the aromatization of testosterone [24]. This model allows for control of circulating estrogen levels in vivo, and is a well-established model for the study of estrogenic modulation [25,26]. Next, using the same gonadectomy model, we characterize the effects of estrogen replacement in the context of either a PTS-inducing noise exposure (102.5 dB sound pressure level [SPL], 8–16 kHz, 2-h) or a less-severe, TTS-inducing noise exposure that causes cochlear synaptopathy (94 dB SPL, 8–16 kHz, 2-h).

Our data indicate that estrogen replacement modulates peripheral auditory physiology in female mice in the absence of noise exposure. We also demonstrate that estrogen replacement in gonadectomized female mice reduces auditory threshold shifts following both a PTS-inducing and TTS-inducing noise exposure. Furthermore, we show that surgical gonadectomy increases, and estrogen replacement decreases, OHC loss and cochlear synaptopathy. Finally, using fluorescent in situ hybridization, we demonstrate colocalization of *Esr2* with type-1C SGNS, suggestive of an E_2_-mediated protective effect against cochlear synaptopathy through preservation of type-1C SGNs. Taken together, these data indicate the estrogen signaling pathways may show promise for the prevention and treatment of NIHL.

## 2. Results

### 2.1. E_2_-Replacement Decreases Auditory Thresholds and Increases Wave-1 Amplitude

**Figure 1:**
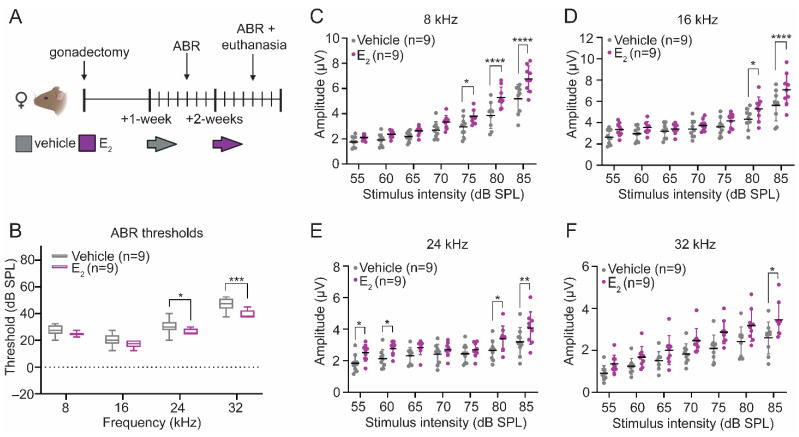
E_2_-replacement in gonadectomized female mice modulates auditory brainstem response (ABR) thresholds and wave-1 amplitudes. (**A**) Schematic of the within-animal experimental design. Female mice were gonadectomized at 8-weeks of age. Mice received 3 daily subcutaneous injections of vehicle 1-week post-gonadectomy and 3 daily subcutaneous injections of E_2_ (300 µg/kg) 2-weeks post-gonadectomy. (**B**) Following E_2_-replacement, ABR thresholds improved at 24 kHz (*p* = 0.0416) and 32 kHz (*p* = 0.0003). (**C**–**F**) E_2_-replacement increases suprathreshold ABR wave-1 amplitudes at (**C**) 8 kHz (75 dB sound pressure level [SPL]: *p* = 0.0358; 80 dB SPL: *p* < 0.0001; 85 dB SPL: *p* < 0.0001); (**D**) 16 kHz (80 dB SPL: *p* = 0.0128; 85 dB SPL: *p* < 0.0001); (E) 24 kHz (55 dB SPL: *p* = 0.0345; 60 dB SPL: *p* = 0.0438; 80 dB SPL: *p* = 0.0134; 85 dB SPL: *p* = 0.0023); and (**F**) 32 kHz (85 dB SPL: *p* = 0.0325). ABR thresholds and wave-1 amplitudes are compared before and after E_2_-replacement in the same animals using a RM 2-way ANOVA followed by Sidak’s multiple comparison test. *n* = number of mice; ABR thresholds: minimum value, 1st quartile, median, 3rd quartile, maximum value; Wave-1 amplitudes: mean ± SD; * *p* < 0.05, ** *p* < 0.01, *** *p* < 0.001, **** *p* < 0.0001.

We quantified auditory brainstem responses (ABRs), which reflect synchronous neural activity along the ascending auditory pathway [27], to evaluate the effects of E_2_-replacement on peripheral auditory physiology in the absence of noise exposure. Eight-week-old female mice underwent surgical gonadectomy and recovered for a period of 1-week. At 9-weeks of age, all mice were administered a vehicle control, and auditory thresholds and wave-1 amplitudes were quantified via ABR measurements. At 10-weeks of age, the same cohort was acutely administered E_2_. The day following the final subcutaneous injection of E_2_, auditory thresholds and wave-1 amplitudes were quantified via ABR measurements (Figure 1A).

**Figure 2:**
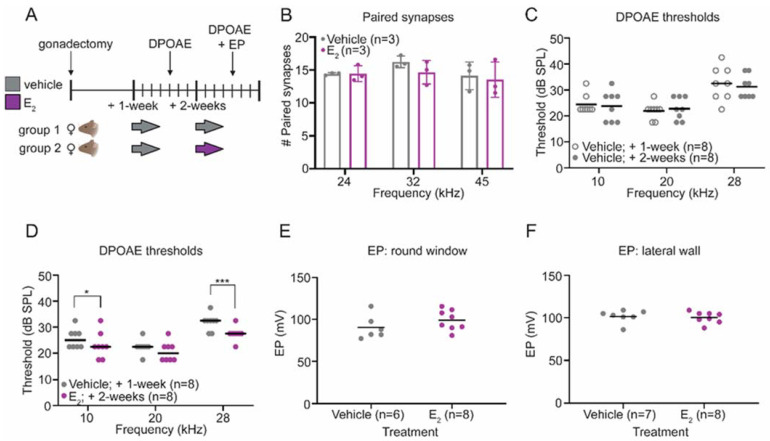
E_2_-replacement in gonadectomized female mice improves distortion product otoacoustic emission (DPOAE) thresholds but does not alter the number of paired inner hair cell (IHC) synapses or the endocochlear potential. (**A**) Schematic of the experimental design. Both cohorts of mice received 3 daily subcutaneous injections of vehicle 1-week post-gonadectomy. Two-weeks post-gonadectomy, one cohort received 3 daily subcutaneous injections of vehicle, while the other cohort received 3 daily subcutaneous injections of E_2_ (300 µg/kg). (**B**) Histological analysis of cochlear tissue collected from vehicle-treated and E_2_-treated mice demonstrated no differences in the number of paired IHC-auditory nerve fiber (ANF) synapses. (**C**) Analysis of DPOAE thresholds in the cohort treated with vehicle during both weeks of data collection revealed no changes. (**D**) Following E_2_-replacement, gonadectomized female mice displayed improved DPOAE thresholds at 10 kHz (*p* = 0.0216) and 28 kHz (*p* = 0.0001). (**E**,**F**) The endocochlear potential measured via a round window approach (**E**) and via a lateral wall approach (**F**) is not changed by E_2_-replacement. DPOAE thresholds were compared within groups using a RM 2-way ANOVA followed by Sidak’s multiple comparison test. Paired synapses were compared using a 2-way ANOVA followed by Sidak’s multiple comparisons test. Endocochlear potentials were compared using a student’s *t*-test. *n* = number of mice; Paired synapses: mean ± SD; DPOAE thresholds and EP measurements: bars display the mean; * *p* < 0.05, *** *p* < 0.001.

Auditory thresholds and wave-1 amplitudes were compared before and after E_2_-replacement within the same animals. Following E_2_-replacement, ABR thresholds improved at 24 kHz (*p* = 0.0416) and 32 kHz (*p* = 0.0003) (Figure 1B). Suprathreshold wave-1 amplitudes—a measure of the synchronous neural activity at the level of the spiral ganglion—were compared before and after E_2_-replacement in each animal. We observed statistically significant increases in the wave-1 amplitudes at each frequency tested, particularly at the higher stimulus intensities (Figure 1C–F). Using an additional cohort, we quantified the number of IHC synapses in E_2_-treated and vehicle-treated female mice to determine if the observed improvements in auditory thresholds and increase in wave-1 amplitudes could be the result of changes to the number of paired IHC-ANF synapses. This cohort utilized a between animal design (Figure 2A). Mice in the vehicle-treated condition received additional subcutaneous vehicle injections during the second week of data collection, while mice in the E_2_-treated condition received subcutaneous injections of E_2_. Like the original cohort, E_2_-treated mice displayed significantly larger wave-1 amplitudes at all frequencies examined (data not shown). Despite the increase in wave-1 amplitudes observed in the E_2_-treated mice, there was no significant effect of treatment on the number of paired synapses per IHC at any frequency tested (Figure 2B), indicating that the observed increase in wave-1 amplitude following E_2_-replacement cannot be attributed to an increase in the number of paired synapses.

### 2.2. E_2_-Replacement Decreases Distortion Product Otoacoustic Emission Thresholds

Within the cochlea, OHCs act as signal amplifiers and contribute to ABRs through their effects on IHCs [28]. Distortion product otoacoustic emissions (DPOAEs) are sounds emitted specifically by OHCs as a result of their electromotive properties [29], and can be used to assess OHC function [30]. To investigate whether the improved auditory thresholds and increase in wave-1 amplitudes may be the result of an increase in signal amplification by OHCs, we quantified DPOAE thresholds in vehicle-treated and E_2_-treated female mice (Figure 2A).

We observed no changes in DPOAE thresholds in mice that received vehicle injections during both weeks of data collection (Figure 2C). In contrast, in the cohort that received E_2_-replacement during the second week of data collection, within-group analysis revealed improved DPOAE thresholds following E_2_-replacement at 10 kHz (*p* = 0.0216) and 28 kHz (*p* = 0.0001), suggesting that E_2_-replacement modulates OHC physiology in gonadectomized female mice in the absence of noise exposure (Figure 2D).

### 2.3. E_2_-Replacementon Does Not Affect the Endocochlear Potential

Another possible explanation for the observed electrophysiological changes, including the improvement in DPOAE thresholds, is that E_2_-replacement modulates the endocochlear potential (EP). The apical surfaces of the mechanosensory cells in the inner ear are bathed in an extracellular fluid called endolymph, normally characterized by a high potassium (K^+^) concentration and a positive electric potential (in mice) of about 100 mV [31,32]. This positive EP is critical for cochlear hair cell mechanotransduction, and changes to the EP can alter ABR thresholds [33,34,35]. The EP was measured in 10-week-old vehicle-treated and E_2_-treated female mice (Figure 2A). This experiment was performed two ways in different cohorts, first using a round window approach (Figure 2E) and second using a lateral wall approach (Figure 2F) to verify consistency in EP measurements between the two experimental techniques. We observed no differences between the vehicle-treated and E_2_-treated animals using either experimental approach, indicating E_2_-replacement does not affect the EP, and that the observed electrophysiological changes following E_2_-replacement are not the result of changes to the EP.

### 2.4. Gonadectomy Eliminates Sex Differences in the Response to PTS-Inducing Noise

**Figure 3:**
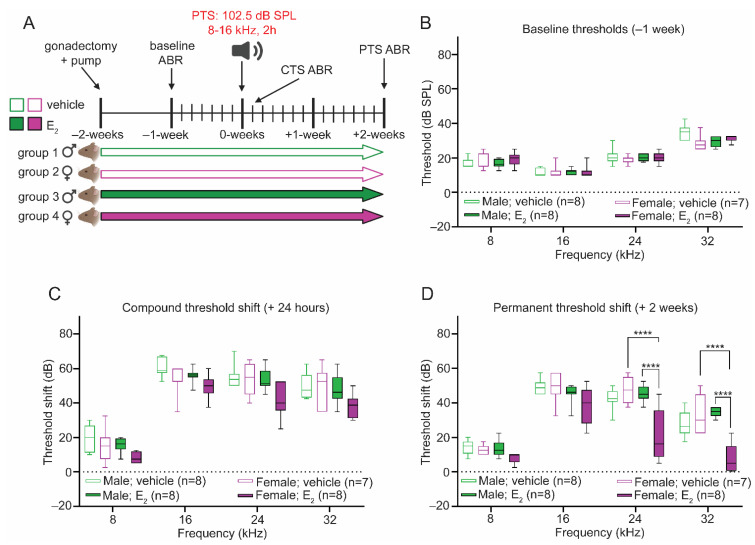
E_2_-replacement in gonadectomized female mice protects against hearing loss following a PTS-inducing noise exposure. (**A**) Experimental schematic. Male and female mice were gonadectomized at 8-weeks of age and implanted with an osmotic pump to deliver vehicle or E_2_ (1 μg/day) for the duration of the study. Animals were exposed to a permanent threshold shift (PTS)-inducing noise (102.5 dB SPL, 8–16 kHz, 2-h) at 10-weeks of age. Auditory thresholds were quantified via ABR measurements 1-week pre-exposure (baseline), 24-h post-exposure (compound threshold shift or CTS), and 2-weeks post-exposure (permanent threshold shift or PTS). (**B**) Vehicle-treated and E_2_-treated male and female mice display similar baseline ABR thresholds. (**C**) Twenty-four hours post-exposure, vehicle-treated and E_2_-treated male and female mice display a CTS of a similar magnitude at all frequencies examined. (**D**) Two-weeks post-exposure, E2-treated female mice display a reduced PTS compared to E_2_-treated males at 24 kHz (*p* < 0.0001) and 32 kHz (*p* < 0.0001) and compared to vehicle-treated females at 24 kHz (*p* < 0.0001) and 32 kHz (*p* < 0.0001). Vehicle-treated male and female mice display a PTS of a similar magnitude across all frequencies tested. Similarly, vehicle-treated and E_2_-treated males displayed no differences in the magnitude of the PTS across all frequencies tested. ABR thresholds were compared using a 3-way ANOVA followed by Tukey’s post-hoc test. *n* = number of mice; ABR thresholds: minimum value, 1st quartile, median, 3rd quartile, maximum value; **** *p* < 0.0001.

Following the evaluation of the effects of E_2_-replacement on peripheral auditory physiology in the absence of noise exposure, we next evaluated the potential protective effects of E_2_-replacement against NIHL. In a previous report, we demonstrated that gonadally intact female B6CBAF1/J mice displayed relative protection from PTS-inducing noise (101 dB SPL, 8–16 kHz, 2-h) in comparison to gonadally intact males [16]. Here, we examined whether the previously documented protection from PTS-inducing noise in female mice is a result of gonadal E_2_. Eight-week-old male and female B6CBAF1/J mice were gonadectomized and implanted with a subcutaneous osmotic pump delivering either E_2_ or a vehicle control (Figure 3A). ABR measurements were used to compare hearing thresholds at 3 timepoints: (1) following gonadectomy and 1-week before noise exposure (baseline); (2) 24-h post-exposure to quantify the compound threshold shift (CTS) [36]; and (3) 2-weeks post-exposure to quantify the PTS (Figure 3A).

At baseline, there were no differences in auditory thresholds between vehicle-treated males and females at any frequency tested (Figure 3B). Twenty-four hours after the PTS-inducing exposure, gonadectomized vehicle-treated male and female mice displayed no differences in the magnitude of the CTS across all frequencies tested (Figure 3C). Similarly, ABR measurements collected 2-weeks post-exposure in gonadectomized vehicle-treated male and female mice displayed no differences in the magnitude of the PTS across all frequencies tested (Figure 3D). These results demonstrate that the sex-dependent protective effect previously reported is abolished in the absence of circulating gonadal steroids. 

### 2.5. E_2_-Replacement Protects against PTS-Inducing Noise in Female Mice

Using the same experimental timeline, in another cohort of mice we then investigated whether E_2_-replacement re-establishes the sex-dependent protective effect [16]. At baseline, E_2_-treated males and females displayed similar ABR thresholds at all frequencies tested. Additionally, there were no differences in baseline thresholds after E_2_-treatment in male or female mice (Figure 3B).

Analysis of the CTS 24-h post-exposure revealed a significant main effect of treatment (F_1,108_ = 17.15) and biological sex (F_1,108_ = 21.04), although a comparison between E_2_-treated females and either E_2_-treated males or vehicle-treated females did not reveal statistically significant reductions in the CTS at any frequency tested. Vehicle-treated and E_2_-treated males displayed a CTS of a similar magnitude at all frequencies tested (Figure 3C). Analysis of the PTS 2-weeks post-exposure revealed that E_2_-treated females displayed a robust reduction in the magnitude of the PTS compared to both vehicle-treated females and E_2_-treated males at 24 kHz (*p* < 0.0001) and 32 kHz (*p* < 0.0001) (Figure 3D). The observed protective effect of E_2_-replacement was confined to female mice, as E_2_-treated and vehicle-treated males displayed a similar PTS at all frequencies tested (Figure 3D).

### 2.6. E_2_-Replacement Reduces OHC Loss following a PTS-Inducing Noise Exposure

**Figure 4:**
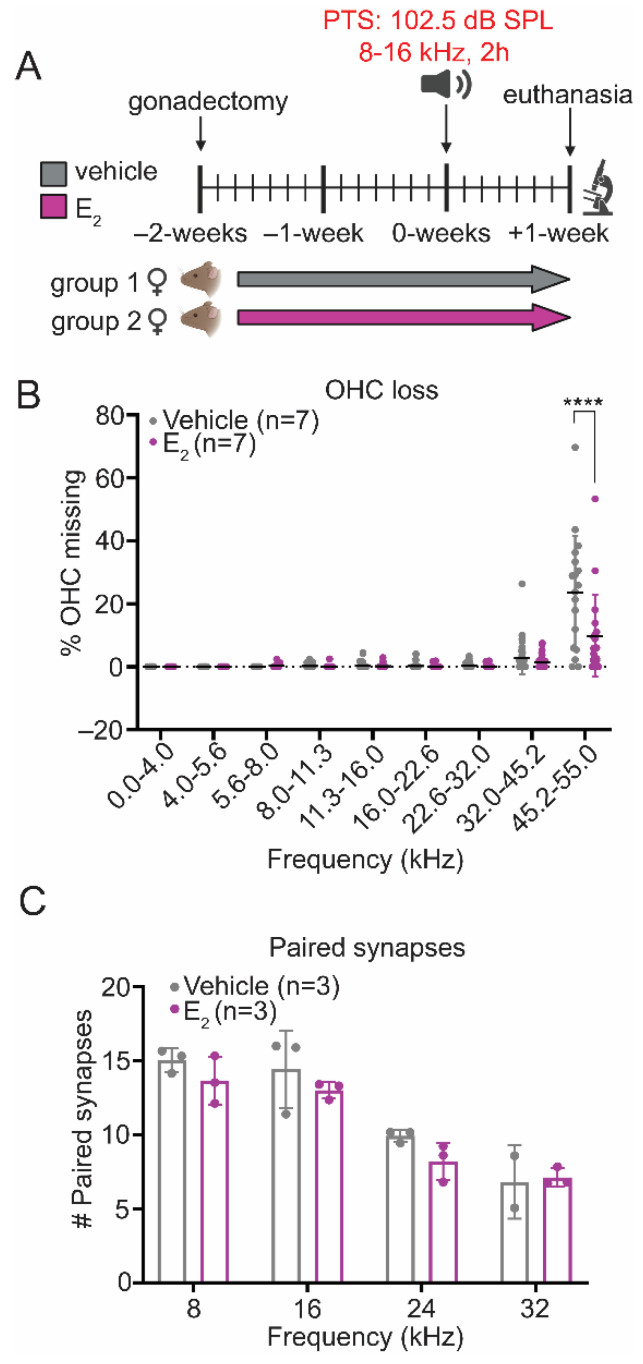
E_2_-replacement in female mice increases outer hair cell (OHC) survival in the base of the cochlea following a PTS-inducing noise exposure. (**A**) Experimental schematic. Female mice were gonadectomized at 8-weeks of age and treated with vehicle or E_2_ (150 µg/kg) beginning 2-days after the gonadectomy for the remaining duration of the study. Animals were euthanized for tissue collection 1-week post-exposure. (**B**) E_2_-replacement in female mice increases OHC survival in the base of the cochlea (45.2–55.0 kHz) 1-week following a PTS-inducing noise exposure (*p* < 0.0001). (**C**) E_2_-replacement in female mice does not reduce the loss of paired IHC-ANF synapses 1-week following a PTS-inducing noise exposure. OHC survival and paired synapse number were compared between groups using a 2-way ANOVA followed by Sidak’s multiple comparison test. *n* = number of ears; plots: mean ± SD; **** *p* < 0.0001.

To validate our physiologic data and to obtain tissue for histological analyses, we utilized an additional cohort where E_2_-replacement was administered using subcutaneous injections of 17β-estradiol-3-benzoate (from here on referred to as E_2_) (Figure 4A). This cohort used female mice only because we observed E_2_-mediated protection from PTS-inducing noise exposure in female but not male mice. Cochleae were collected 1-week following the PTS-inducing noise exposure, since we determined in previous studies that permanent threshold shifts did not change between the 1-week and 2-week post-exposure ABRs [16]. In the areas corresponding to the frequency-specific PTS—16 kHz, 24 kHz, and 32 kHz—there was no OHC loss and therefore no difference in OHC survival between the experimental conditions. However, in the basal portion of the cochlea corresponding to frequencies between 45.2 kHz and 55.0 kHz, E_2_-treated mice displayed significantly improved OHC survival (*p* < 0.0001), consistent with an E_2_-induced otoprotective effect (Figure 4B). The pattern of OHC loss following this noise exposure paradigm is consistent with our previously published data, showing that a similar noise exposure in this strain of mice results in an extensive loss of OHCs in the basal part of the cochlea, whereas the frequency-specific hearing loss is associated with OHC dysfunction but not with an immediate OHC loss [16]. Paired IHC synapses were also quantified from the same cohort of animals 1-week post-exposure. There were no observed differences in paired synapse numbers between the E_2_-treated and vehicle-treated female mice at any frequency examined (Figure 4C).

### 2.7. Protective Effect of E_2_-Replacement on Hearing Thresholds following TTS-Inducing Noise

While E_2_-replacement ameliorated hearing loss and improved OHC survival in female mice following a PTS-inducing noise exposure, vehicle-treated and E_2_-treated female mice displayed a similar degree of synapse loss (compare Figure 2B and Figure 4C). The sound energy required to induce a PTS exceeds the sound energy required to induce cochlear synaptopathy in CBA/CaJ mice [37], and permanent shifts in auditory thresholds are concomitant with synaptic uncoupling and dysfunction to a degree that may have exceeded the therapeutic capacity of E_2_-replacement. Thus, to evaluate the effect of E_2_-replacement on protection from cochlear synaptopathy, we utilized a less-intense, TTS-inducing noise exposure (94 dB SPL, 8–16 kHz, 2-h). Given that the protective effect of E_2_ was confined to female mice in the PTS-inducing experiments, our investigation of E_2_’s protective effects from here on utilized female mice only.

**Figure 5:**
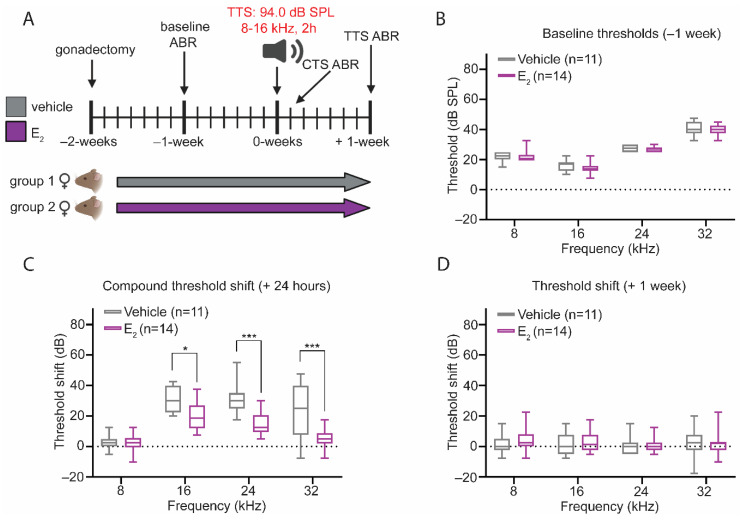
E_2_-replacement in gonadectomized female mice reduces the CTS after a temporary threshold shift (TTS)-inducing noise exposure. (**A**) Experimental schematic. Female mice were gonadectomized at 8-weeks of age and treated with vehicle or E_2_ (150 µg/kg) beginning 2-days after the gonadectomy for the remaining duration of the study. Animals were exposed to a TTS-inducing noise (94 dB SPL, 8–16 kHz, 2-h) at 10-weeks of age. Auditory thresholds were quantified via ABR measurements 1-week pre-exposure (baseline), 24-h post-exposure (CTS), and 1-week post-exposure. (**B**) At baseline, vehicle-treated and E_2_-treated mice display no differences in ABR thresholds at any frequency tested. (**C**) E_2_-treated female mice display a reduced CTS at 16 kHz (*p* = 0.0266), 24 kHz (*p* = 0.0001), and 32 kHz (*p* = 0.0001). (**D**) Despite the differences in the magnitude of the CTS, there are no differences in ABR thresholds 1-week post-exposure between the E_2_-treated and vehicle-treated female at any frequency tested. ABR thresholds were compared using a 2-way ANOVA followed by Tukey’s post-hoc test. *n* = number of mice; ABR thresholds: minimum value, 1st quartile, median, 3rd quartile, maximum value; * *p* < 0.05, *** *p* < 0.001.

Eight-week-old female B6CBAF1/J mice underwent surgical gonadectomy and subsequently received vehicle treatment or E_2_-replacement (Figure 5A). At baseline, there were no differences in auditory thresholds between the vehicle-treated and E_2_-treated mice (Figure 5B). Twenty-four hours following the noise exposure, analysis of the CTS revealed that E_2_-treated mice displayed a significantly reduced CTS at 16 kHz (*p* = 0.0266), 24 kHz (*p* = 0.0001), and 32 kHz (*p* = 0.0001), compared to the vehicle-treated mice (Figure 5C). Consistent with this noise exposure producing a TTS, auditory thresholds returned to baseline in both experimental conditions, and comparison of the 1-week threshold shifts between treatment groups revealed no differences (Figure 5D). 

Next, we evaluated the effect of E_2_-replacement on ABR wave-1 amplitudes. A reduction in wave-1 amplitude in TTS-inducing noise exposure paradigms is used to indirectly assess the presence of cochlear synaptopathy, although recent studies demonstrate the potential for a reduction in wave-1 amplitude also in the absence of cochlear synaptopathy [17].

**Figure 6:**
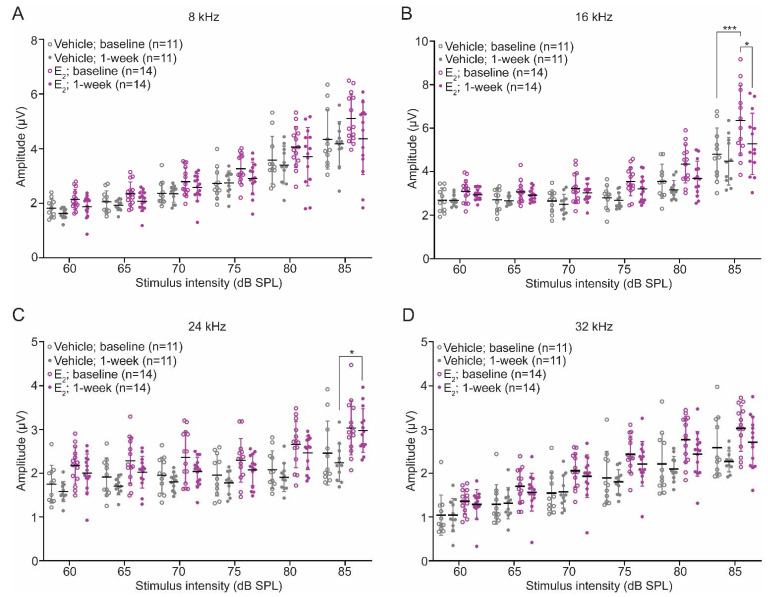
E_2_-replacement in gonadectomized female mice increases wave-1 amplitudes before and after a TTS-inducing noise exposure. (**A**–**D**): Wave-1 amplitudes in vehicle-treated and E_2_-treated mice at baseline and 1-week post-exposure. Female mice were gonadectomized at 8-weeks of age and treated with vehicle or E_2_ (150 µg/kg) beginning 2-days after the gonadectomy for the remaining duration of the study. Animals were exposed to a TTS-inducing noise (94 dB SPL, 8–16 kHz, 2-h) at 10-weeks of age. Wave-1 amplitudes were quantified via ABR measurements 1-week pre-exposure (baseline) and 1-week post-exposure. (**B**) At baseline, E_2_-treated mice display larger wave-1 amplitudes at 16 kHz: 85 dB SPL (*p* = 0.0001). (**C**) One-week post-exposure E_2_-treated female mice display larger wave-1 amplitudes at 24 kHz: 85 dB SPL (*p* = 0.0111). Wave-1 amplitudes were compared using a 3-way ANOVA followed by Tukey’s post hoc test. *n* = number of mice; Wave-1 amplitude: mean ± SD; * *p* < 0.05, *** *p* < 0.001.

**Figure 7:**
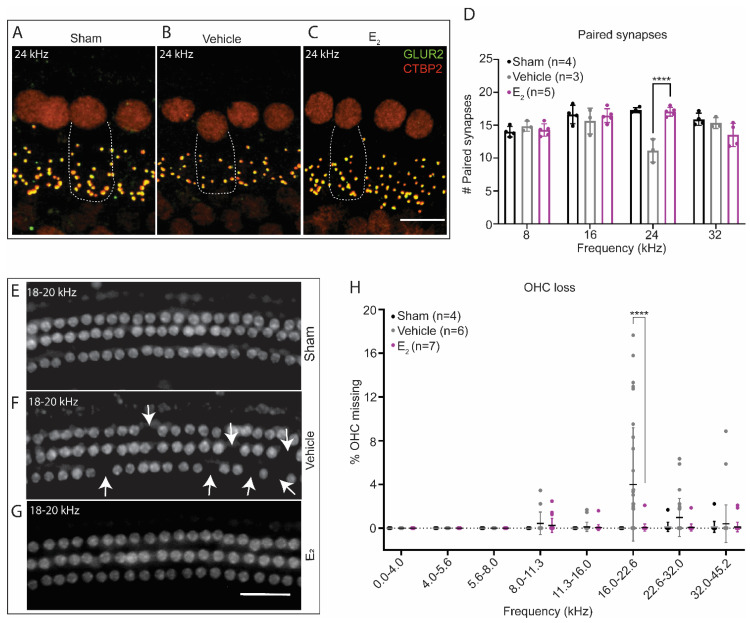
E_2_-replacement in gonadectomized female mice ameliorates cochlear synaptopathy and OHC loss following a TTS-inducing noise exposure. (**A**–**C**) Representative images of paired IHC-ANF synapses at 24 kHz 1-week post-exposure in (**A**) sham-exposed mice, (**B**) vehicle-treated mice, and (**C**) E_2_-treated mice. Paired synapses were identified via the co-localization of CTBP2 (red) and GLUR2 (green). Sham animals were gonadectomized and vehicle-treated but not noise-exposed. (**D**) E_2_-replacement (150 μg/kg) ameliorates cochlear synaptopathy at 24 kHz 1-week following a TTS-inducing noise (94 dB SPL, 8–16 kHz, 2-h) (*p* < 0.0001). Compared to a group of sham exposed mice, E_2_-treated mice display no loss of synapses at 24 kHz, while vehicle-treated mice display, on average, a loss off 5.9 synapses at this frequency. (**E**–**G**) Representative cytocochleograms 1-week post-exposure at the approximate cochlear location of 18–20 kHz in (**E**) sham-exposed mice, (**F**) vehicle-treated mice, and (**G**) E_2_-treated mice. White arrows indicate missing OHCs. (**H**) Vehicle-treated mice display increased susceptibility to OHC loss 1-week following a TTS-inducing noise exposure. Compared to E_2_-treated female mice, vehicle-treated female mice displayed significantly increased OHC loss restricted to the frequencies between 16.0 kHz and 22.6 kHz (*p* < 0.0001). Neither group of animals displayed significant OHC loss below 16 kHz or above 22.6 kHz. Paired synapse counts and OHC loss were compared using a 2-way ANOVA followed by Sidak’s multiple comparison test. Dotted lines in (**A**–**C**) represent the outline of a single inner hair cell. *n* = number of ears, plots: mean ± SD; scale bar in (**C**): 10 µm; scale bar in (**G**): 50 µm; **** *p* < 0.0001.

Wave-1 amplitudes were extracted from ABR tracings at baseline and 1-week post-exposure. Analysis of baseline amplitudes revealed a significant main effect of treatment at each frequency tested, indicating E_2_-treated mice displayed significantly larger ABR wave-1 amplitudes overall compared to vehicle-treated mice (Figure 6A–D, Table A1). One-week post-exposure, wave-1 amplitudes decreased in both groups of mice but remained larger overall in the E_2_-treated mice, as a comparison of wave-1 amplitudes revealed a significant main effect of treatment at all frequencies tested (Figure 6A–D). The maintenance of larger wave-1 amplitudes in the E_2_-treated mice suggests increased synchronous neural activity at the level of the spiral ganglion and potentially an E_2_-mediated protection from cochlear synaptopathy. We therefore quantified the number of paired synapses in vehicle-treated and E_2_-treated mice 1-week post-exposure. Compared to a group of sham exposed mice, E_2_-treated mice displayed the same number of synapses at each frequency examined. In contrast, compared to the E_2_-treated mice, vehicle-treated mice displayed an average loss of 5.9 paired synapses at 24 kHz (*p* < 0.0001), suggestive of a frequency-dependent, E_2_-mediated protective effect from cochlear synaptopathy following a TTS-inducing noise exposure (Figure 7A–D).

### 2.8. Gonadectomy Increases Susceptibility to OHC Loss

Outer hair cell loss is typically not a pathophysiological outcome of exposure to TTS-inducing noise [12,38,39]. However, the effect of TTS-inducing noise on OHC survival has not been previously compared between intact and gonadectomized mice. We therefore performed cytocochleograms to examine OHC survival in the same cohort of animals 1-week post-exposure. As expected, E_2_-treated mice did not display any loss of OHC. In contrast, compared to E_2_-treated mice, vehicle-treated mice displayed a 3.9% increase in OHC loss (*p* < 0.0001) 1-week post-exposure that was restricted to frequencies between 16.0 kHz and 22.6 kHz (Figure 7E–H). Like the E_2_-treated mice, vehicle-treated mice did not display significant OHC loss below 16.0 kHz or above 22.6 kHz. 

### 2.9. Canonical Estrogen Receptor Localization

**Figure 8:**
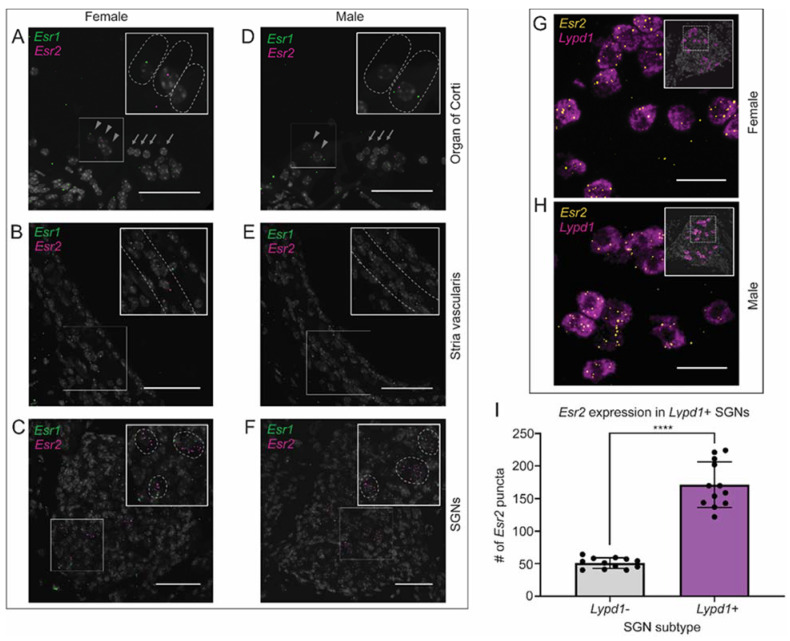
Fluorescent in situ hybridization of estrogen receptor 1 (*Esr1*) and estrogen receptor 2 (*Esr2*). mRNA expression in the organ of Corti, stria vascularis, and spiral ganglion neurons of both male and female mice (**A**–**F**). *Esr1* (green puncta) and *Esr2* (magenta puntca) are present at low levels in the inner hair cells of the organ of Corti, marked by grey arrowheads, but are absent in the outer hair cells, marked by grey arrows (**A**,**D**). *Esr1* is expressed throughout the three cell layers of the stria vascularis, while *Esr2* expression appears highest in the basal cell layer, marked by dashed grey lines (**B**,**E**). *Esr1* expression is low and diffuse within the spiral ganglion neurons, while *Esr2* expression appears highest in a specific neuronal subtype (**C**,**F**). Scale bars: 50 μm. (**G**,**H**) *Esr2*, represented by yellow punctate dots, is co-expressed with *Lypd1*, a type-1C SGN marker seen in magenta. (**I**) *Esr2* expression is highest in type-1C SGNs compared to any other cell type in the spiral ganglion (**** *p* < 0.0001; student’s *t*-test). Scale bars: 25 μm.

To identify possible mechanisms through which estrogen alters auditory physiology and confers protection against NIHL, we characterized the expression of estrogen receptors 1 and 2 (*Esr1*, *Esr2*) transcripts in male and female mice. Fluorescent in situ hybridization RNAscope™ assays were performed to localize the receptor transcripts to specific cell types in the cochlea, with representative images taken from the middle turn of the cochlea (Figure 8A–F). No sex differences in the localization of either *Esr1* or *Esr2* were detected. In the organ of Corti, expression of *Esr1* and *Esr2* was observed at low levels in the IHCs, whereas the OHCs demonstrated no expression of either receptor (Figure 8A,D). Moderate levels of *Esr1* and *Esr2* expression were seen within the stria vascularis (SV), with *Esr1* similarly distributed throughout the three cell layers comprising the SV (Figure 8B,E). Distribution of *Esr2* mRNA appeared more highly expressed in the basal cell layer compared to the marginal and intermediate cells. In contrast to the organ of Corti and stria vascularis, the most abundant expression of estrogen receptors was observed within the SGNs (Figure 8C,F). Interestingly, while *Esr1* expression was lower and diffuse throughout the SGNs, *Esr2* exhibited robust and highly specific expression in a subset of neurons. 

To determine the identity of this SGN subtype, additional fluorescent in situ hybridization assays were performed using a probe for *Lypd1*, an type-1C SGN marker, in combination with a probe for *Esr2* [10]. In both male and female mice, we found significantly greater co-expression of *Lypd1* and *Esr2* together versus *Esr2* alone (*p* < 0.0001), suggesting *Esr2* localization is primarily confined to the type-1C SGNs in the adult mouse cochlea (Figure 8G–I).

## 3. Discussion

Our laboratory previously identified biological sex as a critical factor that influences the severity of hearing loss following noise exposure and showed that adult female mice are protected in comparison to males [16]. Here, we used a gonadectomy model to demonstrate that gonadal E_2_ underpins, at least partially, the relative protection in female mice. In the absence of endogenous gonadal hormones, we demonstrated that males and females are equally susceptible to noise exposure. Our data show that E_2_-replacement in female mice ameliorates threshold shifts following PTS- and TTS-inducing noise exposures and reduces loss of OHC and paired synapses. Furthermore, to the best of our knowledge, our data reveal, for the first time, colocalization of *Esr2* and type 1-C SGNs and suggest that protection from cochlear synaptopathy may occur through E_2_-mediated preservation of these neurons.

E_2_-replacement increased OHC survival in female mice following both types of noise exposures. To our surprise, surgical gonadectomy without E_2_-replacement resulted in a focal loss of OHCs between 16.0 kHz and 22.6 kHz 1-week following a TTS-inducing noise exposure. Frequency-specific OHC loss has not generally been reported for any animal model following a TTS-inducing exposure [12,38,39], nor was frequency-specific OHC loss observed in intact female mice of the same strain (B6CBAF1/J) following a more intense PTS-inducing noise exposure [16]. These results suggest that OHCs in females are uniquely vulnerable to noise exposure in the absence of baseline levels of circulating gonadal steroid hormones and are prone to cell death even following less-intense noise exposures. The potential translational significance of this finding for post-menopausal women is supported by additional evidence demonstrating that the decrease in E_2_ levels associated with menopause prompts a decline in hearing sensitivity [40,41].

In mice exposed to TTS-inducing noise, E_2_-replacement prevented cochlear synaptopathy at 24 kHz, the frequency with the maximal CTS in our animal model. The observed protection from synaptopathy may occur via prevention of the uncoupling of the paired IHC-ANF synapses, synaptic regeneration, or a combination of both phenomena. Recent studies demonstrate the potential for synaptic regeneration as treatment with bisphosphonates 24-h post-exposure can reverse synaptopathy [42]. Histological evaluation at additional post-exposure timepoints is needed to discriminate the two proposed mechanisms. 

Our data also demonstrate that E_2_-replacment is sufficient to modulate peripheral auditory physiology in the absence of noise exposure. In particular, we observed a robust effect of E_2_-replacement on wave-1 amplitude, where E_2_-treated female mice displayed larger wave-1 amplitudes at all frequencies examined despite no differences in the number of paired IHC-ANF synapses. An increase in wave-1 amplitudes in the absence of changes to paired synapse numbers could arise from the non-genomic actions of estrogens, which include the regulation of calcium signaling and cyclic adenosine monophosphate (cAMP). Previous studies demonstrate that estrogens potentiate L-type calcium channels like CaV1.3, which is found at the inner hair cell synapse [43,44]. An increase in cAMP and CaV1.3 conductance could lead to an increase in glutamate release [45]. The increase in glutamate release, in turn, could lead to an increase in the speed, or number, of spike generations and cause an increase in wave-1 amplitude [46]. Further functional studies are required to investigate this hypothetical mechanism.

E_2_ is a known ligand of the two classical estrogen receptors, ESR1 and ESR2. Upon binding with E_2_, ESR1 and ESR2 homo- and hetero-dimerize and translocate to the nucleus where they regulate transcription of target genes [19,47]. Previous reports suggest that ESR1 and ESR2 are both expressed in the cochlea of CBA inbred strains [48,49] and that protein levels of ESR2 may be expressed at a higher level than ESR1 in the inner ear SGNs [48]. Furthermore, ESR2 has been functionally implicated in ARHL and NIHL [22,23]. Our data demonstrate localization of both *Esr1* and *Esr2* transcripts in the SGNs of intact adult male and female mice. However, we also show that *Esr2* localizes more specifically to type-1C SGNs. These data provide additional evidence to support our hypothesis that the protective effects of estrogen-signaling following noise exposure—in this case, the amelioration of cochlear synaptopathy following noise trauma—may be mediated via ESR2. Previous studies have suggested that synaptopathy-inducing noise results specifically in the retraction of type-1C fibers, suggesting E_2_-replacement protects against the loss of type-1C SGNs that characterizes synaptopathy [9,10]. In support of the histologic data that suggest SGNs are responsive to E_2_-treatment, we show that unexposed E_2_-treated female mice display increased ABR wave-1 amplitudes at all frequencies tested. Future studies should ask why type-IC SGNs may be protected in the presence of E_2_.

In contrast to the robust expression detected in the SGNs, we detected no expression of *Esr1* or *Esr2* transcripts in the OHCs. A previous report demonstrated faint immunostaining of ESR1 and negative immunostaining of ESR2 in the OHCs of CBA mice [49]. Nevertheless, E_2_-treated mice displayed improved DPOAE thresholds—a proxy for OHC function—in the absence of changes to the endocochlear potential. These data suggest that the effect of E_2_ on OHC physiology is not likely to be mediated via the canonical estrogen receptors ESR1 or ESR2 and may be modulated via another estrogen receptor such as GPER1, the estrogen-related receptors (ESRRα, ESRRβ, and ESRRγ), through a more complex interaction between the classical and related receptors [50], or through increased activation of the projections of the medial olivocochlear bundle [51].

We report, for the first time, an E_2_-mediated protection against cochlear synaptopathy following noise exposure. The literature provides examples of E_2_-mediated regulation of synaptic plasticity elsewhere [52,53,54]. In the central nervous system (CNS), E_2_-signaling—particularly through the classical estrogen receptor ESR2—has been shown to enhance synaptic plasticity through an increase in expression of TRKB and BDNF [55]. In the cochlea, TRKB and BDNF have been shown to prevent neuronal retraction following noise exposure—the currently known pathophysiology to underpin cochlear synaptopathy [56,57].

The role of gonadal E_2_ as a neuroprotectant and neuromodulator is also well-established [58,59]. Ovariectomy of female mice induced an increase in amyloid beta plaques in a model of Alzheimer’s disease, while E_2_-replacement partially ameliorated this effect [60]. Additional studies demonstrated that surgical ovariectomy without E_2_-replacement increases vulnerability of hippocampal neurons in a model of glutamatergic excitotoxicity, and that prophylactic E_2_-replacement prevented this vulnerability [61]. Of significance, glutamatergic excitotoxicity is thought to be a key mediator of noise-induced cochlear synaptopathy [62,63,64]. Taken together, our data show an E_2_-mediated otoprotective effect from noise-induced synaptopathy that is consistent with the role of E_2_-signaling in the CNS and likely mediated via ESR2. These findings are clinically significant, as ESR2-mediated signaling is non-feminizing, and FDA-approved ESR2-modulators are already available [65,66].

Our data further indicate that the otoprotective effects of gonadal E_2_ against PTS-inducing noise exposure may be confined to females, and that females and males are equally susceptible to PTS-inducing noise when devoid of their gonadal hormones. Additional investigation is needed to determine whether E_2_ is protective against TTS-inducing noise in male mice. Consistent with our findings, in a model of Parkinson’s disease, E_2_-replacement in gonadectomized female rats conferred neuroprotection, while E_2_-replacement in gonadectomized males proved deleterious [67]. Conversely, in other models, E_2_-replacement has shown beneficial effects in gonadectomized rats of both sexes. E_2_-replacement has shown to suppress the increase in brain mitochondrial oxidative stress following gonadectomy [68] and confer neuroprotection in an experimental model of stoke [69,70]. Taken together, these reports indicate a tissue-specific interaction between biological sex and the neuroprotective effects of gonadal E_2_.

The system-specific similarities and differences in the neuroprotective actions of E_2_ in males and females are likely due to a combination of organizational and activational effects. Organizational effects arise from changes during the developmental hard wiring of the brain, whereas activational effects arise from modulation by circulating gonadal hormones [59,71]. In support of an organizational component, both male and female ESR2 KO mice show increased susceptibility to TTS-inducing noise [22]. Additional evidence for an organizational component in the auditory system arise from studies of ESRRα, ESRRβ, and ESRRγ. These receptors do not bind E_2_, but nevertheless regulate many of the same transcriptional targets as ESR1 and ESR2 [50]. At least one identified human polymorphism in ESRRβ predicts audiometric TTS in musicians of both sexes, while additional polymorphisms in ESRRβ and ESRRγ cause non-syndromic, congenital hearing impairment and are also implicated in the maintenance of hearing [72,73,74,75]. On the other hand, our data suggest E_2_ also influences susceptibility to/protection from NIHL via activational effects. Elimination of gonadal steroid hormones via surgical gonadectomy abolishes—and E_2_-replacement re-establishes—an innate relative protection from PTS-inducing noise in adult female mice in comparison to males. Additional evidence in humans demonstrating changes in hearing sensitivity and DPOAE amplitudes in pre- and post-menopausal women also implicate activational effects of E_2_-signaling in the modulation of auditory physiology [76,77].

The findings presented here are immediately clinically relevant, not only for the treatment of NIHL, but also for the treatment of other diseases of the auditory and vestibular system, such as Meniere’s Disease, in which E_2_ levels can correlate with symptom severity [77]. The characterization of sex-differences in NIHL and ARHL—which share common mechanisms [78,79]—figures to bear heavily on the development of new therapeutics. In fact, previous reports already suggest that experimental NIHL therapeutics may exhibit sex-dependent efficacy [16,17]. Furthermore, existing FDA-approved, tissue-specific ESR2 modulators, like raloxifene (currently approved for treatment of osteoporosis in post-menopausal women), have been shown to confer neuroprotective effects in a mouse model of epilepsy [80], rendering such drugs as potential candidates for protection of hearing.

## 4. Materials and Methods

### 4.1. Animals

All animals utilized in the experiments were B6CBAF1/J mice (Jackson Laboratory, Bar Harbor, ME, USA; Stock No 100011). These mice are F1 progeny from a cross between C57BL/6J and CBA/J, which eliminates the influence of strain-specific recessively inherited traits such as resistance or susceptibility to ARHL, NIHL, or sound-induced seizures [16]. The animal facility is controlled for temperature and humidity and has a 12-h light/dark cycle (lights on at 7 a.m.). Mice were provided with dry food and water ad libitum. All surgical and experimental procedures took place during the animals’ light phase. Animals were euthanized by CO_2_ asphyxiation or terminal perfusion with 4% paraformaldehyde and then decapitated.

DPOAE testing and lateral wall EP measurement were completed at the Washington University School of Medicine in St. Louis using a separate cohort of animals. Round window EP measurements were completed at the National Institute on Deafness and other Communication Disorders using a separate cohort of animals. All other animal experiments were completed at the University of Maryland School of Medicine. No animals were transported between institutions at any time.

### 4.2. Gonadectomy

Mice were gonadectomized at 8-weeks of age. Anesthesia was achieved with isoflurane gas. In the female mice, bilateral ovariectomy was performed by making a 5 mm, longitudinal, dorsal incision through the skin and muscle wall. The ovary was separated from the uterus lateral to the oviduct using electrocautery, and the uterine horn was repositioned in the abdominal cavity. The muscle wall was closed using a 4–0 poliglecaprone suture (MWI Animal Health, Boise, ID, USA), and the skin was closed using a 4–0 nylon suture (MWI Animal Health, ID). The same procedure was then repeated on the contralateral ovary. In the male mice, bilateral gonadectomy was performed by making a single 2–3 mm, longitudinal, ventral incision through the scrotum. The testes were separated from the seminal vesicles with electrocautery. The skin was closed as described above. Incision sites were treated with a 2.5% lidocaine and 2.5% Prilocaine Cream (Akorn, Lake Forest, IL, USA) and Bacitracin Zinc antibiotic ointment (Trifecta Pharmaceuticals, Fort Lauderdale, FL, USA). Carprofen (0.05 mg/kg) was administered by subcutaneous injection immediately before the surgical procedure and 24-h post-operatively for analgesia. 

### 4.3. Subcutaneous Pump Placement

Eight-week-old male and female mice underwent surgical gonadectomy as described above. During the procedure, animals were implanted with the Alzet 1004^®^ micro-osmotic pump (DURECT Corporation, Cupertino, CA, USA). In female mice, pumps were implanted subcutaneously via the incision created during the ovariectomy. In male mice, a 1 cm, dorsal, longitudinal incision was made between the scapulae. To offset the position of the pump relative to the incision, a subcutaneous pocket was developed by blunt dissection lateral to the incision. All animals recovered for 5–7 days prior to the baseline ABR.

### 4.4. Hormonal Treatments

We utilized three hormonal regimens to generate the data presented in this manuscript. In our experiments evaluating the acute effects of hormone replacement on auditory physiology in the absence of noise exposure, we utilized a series of daily subcutaneous injections administered over a period of 3–4 days. Mice in the NIHL experiments received continuous hormonal replacement for the duration of the studies, a period of 3–4 weeks, which was achieved using either implanted subcutaneous pumps or serial subcutaneous injections. Pumps were utilized in the initial NIHL experiments. Subsequent experiments utilized serial subcutaneous injections, which provided greater control and increased confidence in successful hormone delivery.

#### 4.4.1. Acute Hormonal Supplementation Using Subcutaneous Injections

Mice were administered daily subcutaneous injections of E_2_ (Sigma-Aldrich, St. Louis, MO, USA) (300 µg/kg) reconstituted in sesame oil (vehicle) (Sigma-Aldrich)—or vehicle only—for a period of 3–4 days. This dose was chosen to acutely elevate serum E_2_ to supraphysiologic levels prior to electrophysiological evaluation of baseline auditory function.

#### 4.4.2. Continuous Hormonal Supplementation Using Osmotic Pumps

**Figure A1:**
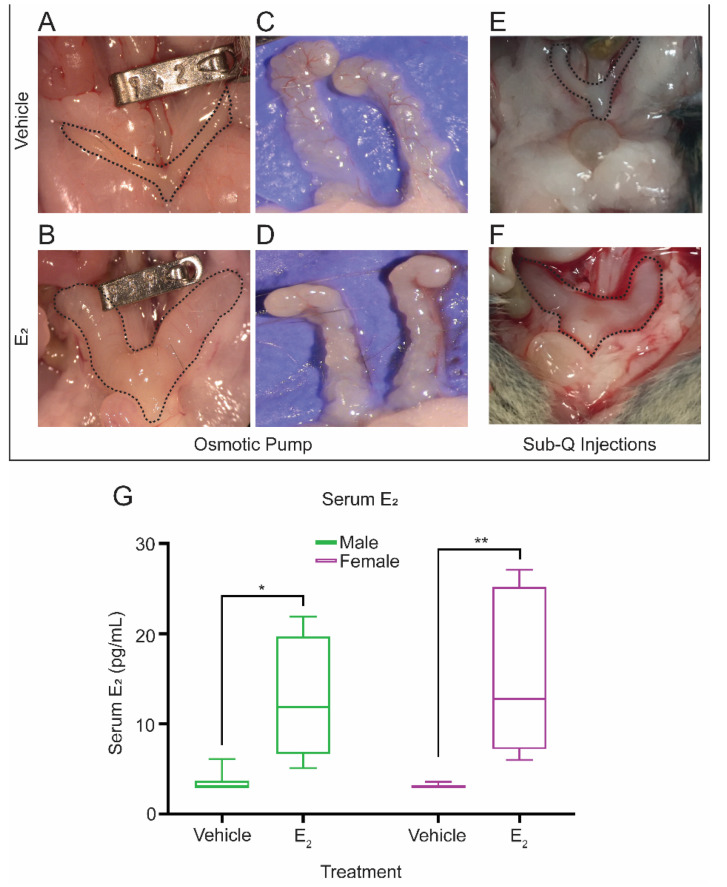
Qualitative and quantitative assessment of E_2_ delivery. (**A**–**F**) Representative photographs of the uterine horns and seminal vesicles of gonadectomized female and male mice administered either vehicle or vehicle/E_2_. The left two columns depict qualitative changes to uterine horns (**A**,**B**) and seminal vesicles (**C**,**D**) with E_2_-replacement delivered via an osmotic pump, while the right column depicts qualitative changes to the female uterine horns (**E**,**F**) when E_2_-replacement is administered via subcutaneous injections. (**G**) Quantitative assessment of serum E_2_ levels collected from the trunk blood of vehicle-treated males (*n* = 8) and females (*n* = 7) and E_2_-treated males (*n* = 8) and females (*n* = 8) when treatments were administered via an osmotic pump. Serum estradiol levels were compared using a 2-way ANOVA (treatment × biological sex) followed by Tukey’s post-hoc test. Box plots: minimum value, 1st quartile, median, 3rd quartile, maximum value; * *p* < 0.05, ** *p* < 0.01.

The Alzet^®^ 1004 micro-osmotic pump was loaded with either 17β-estradiol (E_2_) (Sigma-Aldrich) at a concentration of 38 µg/100 µL in propylene glycol (PPG) or PPG only. The pump delivered 0.11 µL/h of the loaded solutions at a steady release rate to administer a dose of 1 µg of E_2_/day. Drug administration began immediately after pump implantation and continued the entire duration of the study. Serum E_2_ delivery was confirmed qualitatively and quantitatively at the conclusion of the study, four weeks after implantation (Figure A1). Following euthanasia via CO_2_ asphyxiation, the female uterine horns and the male seminal vesicles were exposed and photographed for comparison (Figure A1). Trunk blood was collected, and the sera were processed for estradiol levels as measured via ELISA (ES180S-100, CalBiotech, El Cajon, CA, USA) performed at University of Virginia Ligand Assay and Analysis Core) (Figure A1).

#### 4.4.3. Continuous Hormonal Supplementation Using Subcutaneous Injections

Two to 3 days following surgical gonadectomy, mice were administered subcutaneous injections of 17β-estradiol-3-benzoate (EB) (Sigma-Aldrich) at a concentration of 150 µg/kg reconstituted in sesame oil (vehicle) (Sigma-Aldrich)—or the vehicle only—every other day for the duration of the study. EB is a synthetic pro-drug ester of 17β-estradiol that is de-esterified by nonspecific steroidal esterases to produce biologically active E_2_ in vivo [81,82]. EB delivery is a commonly used method of E_2_ replacement that permits administration of subcutaneous injections every other day rather than every day due to its longer half-life relative to E_2_. Mice that received E_2_-replacement via injections of EB will, from hereon, be described as E_2_-treated or as having received E_2_-replacement following euthanasia, the female uterine horns were exposed and photographed for comparison to confirm successful E_2_ replacement (Figure A1). 

### 4.5. Noise Exposures

Noise exposures were performed using the methods previously described [16]. All mice underwent calibrated noise exposure at 10-weeks of age at the same time of day (8:00 am). Octave-band noise centered at 11.3 kHz (8–16 kHz) was delivered for 2-h using the Fostex FT17H tweeter (Fostex, Tokyo, Japan). Noise was delivered at 102.5 dB sound pressure level (SPL) to induce a PTS or at 94 dB SPL to induce a TTS with cochlear synaptopathy. Calibration was achieved with a measurement microphone (PCB Piezotronics, Depew, NY, USA) placed at the same distance from the tweeter as the mouse’s ears. The tweeter was placed 20 cm above the mice and the sound level was measured to be within 0.5 dB of the target level. During the exposure, mice were placed in a custom holder constructed of perforated aluminum sheets (18 × 15 × 5 cm) with eight equal sized chambers (4.5 × 7.5 × 5 cm) in an acoustically controlled box (IAC Acoustics, North Aurora, IL, USA). Only the four central compartments were used to expose a maximum of four mice simultaneously. The animals were awake and unrestrained throughout the exposure.

### 4.6. Auditory Brainstem Response (ABR)

ABRs were performed using methods described previously [16]. ABRs were recorded at baseline (5–7 days pre-exposure), 24-h after the noise exposure to quantify the compound threshold shift (CTS), and 1–2 weeks after the noise exposure to quantify the permanent threshold shift (PTS) or to confirm a temporary threshold shift (TTS). Animals were anesthetized using an intraperitoneal injection of ketamine (100 mg/kg) (Vet One, Boise, ID, USA) and xylazine (20 mg/kg) (Akorn, Lake Forest, IL, USA). Using the RZ6 recording system (Tucker-Davis Technologies (TDT), Alachua, FL, USA), hearing thresholds were determined at 8 kHz, 16 kHz, 24 kHz, and 32 kHz in an acoustically controlled box (IAC Acoustics, IL). Once surgical levels of anesthesia were achieved, animals were positioned so that they directly faced the tweeter with their ears 10 cm from the front of the tweeter. Subcutaneous electrodes were placed at the post-auricular area of the left and right ears, and at the vertex of the skull for the reference. The ground electrode was placed at the base of the tail. Output stimuli were calibrated with a measurement microphone (PCB Piezotronics, NY) placed at the same distance from the tweeter as the mouse’s ears (10 cm). Frequency-specific tone bursts 2.5 milliseconds (ms) long, with a 0.5 ms sinusoidal on- and off-ramp were delivered with alternating polarity beginning at 90 dB SPL. Tone bursts were progressively decreased by 5 dB until 10 dB below the measurable hearing threshold for each mouse. Electrophysiologic responses to each tone stimulus, filtered between 300 Hz and 3000 Hz, were recorded for 10 ms starting at the onset of the tone, with a total of 512 sweeps at a rate of 21 sweeps/second, and then averaged at each sound level and frequency tested. The responses from each ear were recorded simultaneously. The hearing threshold was defined as the lowest level at which either ABR waves 1 or 2 could be identified. Thresholds were averaged between the two ears and reported as a single value per animal.

Suprathreshold wave-1 amplitudes were extracted offline according to methods described previously [16]. Peak-to-trough wave-1 amplitude was calculated using the maximum positive deflection after 1 ms in the recording and the subsequent maximum negative deflection. Wave-1 amplitudes were averaged between the two ears and reported as a single value per animal.

### 4.7. Distortion Product Otoacoustic Emissions (DPOAE)

DPOAE data (2f1–f2) were collected at the Washington University School of Medicine in St Louis. All recordings were from the left ear. Animals were anesthetized using ketamine and xylazine (80/15 mg/kg, intraperitoneally) and positioned dorsally in a custom headholder. Body temperature was monitored throughout testing using a rectal probe and maintained at 37.5 ± 1.0 °C using a DC current-based isothermal pad (Frederick Haer, Bowdoin, ME, USA). DPOAE input/output relations were obtained for f2’s of 10, 20, and 28.3 kHz using Otoacoustic Emission Average (EMAV) (Boys Town National Research Hospital, Boys Town, NE, USA) in conjunction with TDT and custom hardware. f1 frequencies were given by f2/1.2. The level of f1 (L1) varied from 10 to 90 dB SPL in 5 dB steps while the level of f2 (L2) varied as L1–10 dB. Stimuli were delivered to the ear using a custom coupler inserted using an operating scope. Each channel was output to a TDT EC-1 speaker. DPOAE responses were recorded using a Knowles FC-23652-P16 microphone calibrated to 40 kHz and digitized at 192 kHz. DPOAE thresholds were defined as the minimum sound level required to produce a response of at least −5 dB SPL, well above typical noise levels of about −20 dB SPL. When sound levels straddled this response size, the threshold was taken to be the midpoint.

### 4.8. Endocochlear Potential

#### 4.8.1. Round Window

Data were collected at the National institute on Deafness and Other Communication Disorders in Bethesda, MD. Methods for endocochlear potential (EP) measurement from the round window have been described previously [83,84,85] Here, mice were anesthetized with 2,2,2-tribromoethanol (T4842, Sigma-Aldrich, St. Louis, MO, USA) at a dose of 0.35 mg/g body weight. EP measurements were made using glass microelectrodes inserted into the round window and through the basilar membrane of the first turn of the cochlea. Induction of anoxia, allowing measurement of anoxic-state EP, was accomplished by intramuscular injection of succinylcholine chloride (0.1 µg/g, NDC-0409-6629-02, Pfizer, New York, NY, USA) after establishment of deep anesthesia followed by additional injection of 2,2,2-Tribromoethanol (T4842, Sigma-Aldrich, St. Louis, MO, USA). Anoxic-state EP provides an indicator of passive sensory hair cell polarization. In the presence of functional hair cells, the anoxic-state EP is negative, whereas the EP is zero if the hair cells are not functional. Data were recorded digitally (Digidata 1440A and AxoScope 10; Molecular Devices, San Jose, CA, USA) and analyzed using Clampfit10 (RRID: SCR_011323, Molecular Devices, San Jose, CA, USA). EP measurements were obtained from gonadectomized female mice that received subcutaneous injections of vehicle (*n* = 6 mice) or E_2_ (*n* = 8 mice) as described above with the examiner blinded to treatment received.

#### 4.8.2. Lateral Wall

Data were collected at the Washington University School of Medicine in St Louis. Methods for EP measurement via the lateral wall were performed as previously described [86]. Briefly, animals underwent a single terminal EP measurement from the cochlear lower basal turn of the left ear. Animals were anesthetized (60 mg/kg sodium pentobarbital, IP) (Hospira Incorporated, Lake Forest, IL, USA) and positioned ventrally in a custom headholder. Core temperature was maintained at 37.5 ± 1.0 °C using a thermostatically controlled heating pad in conjunction with a rectal probe (Yellow Springs Instruments Model 73A) (YSI Incorporated, Yellow Springs, OH, USA). An incision was made along the midline of the neck and soft tissues were blunt dissected and displaced laterally to expose the trachea and left bulla. A tracheostomy was then made and the musculature over the bulla was cut posteriorly to expose the bone overlying the round window. Using a fine drill, a hole was made in the left cochlear capsule directly over scala media of the lower basal turn using strial pigment as a guide. Glass capillary pipettes (20–40 MΩ) filled with 0.15 M KCl were mounted on a hydraulic microdrive (Frederick Haer, ME) and advanced until a stable positive potential was observed that did not change with increased electrode depth. The signal from the recording electrode was led to an AM Systems Model 1600 intracellular amplifier (A-M Systems, Sequim, WA, USA). A silver/silver chloride ball inserted into the neck muscles served as ground.

### 4.9. Immunostaining

Immunostaining was performed as previously described [16] with minor modifications. Briefly, tissue was fixed by transcardial perfusion of 4% paraformaldehyde (PFA, Alfa Aesar, Haverhill, MA, USA), followed by harvest of the temporal bones and further fixation in 4% PFA at 4 °C overnight. After adequate decalcification by incubation in 500 mM EDTA (3–6 days), the cochlear ducts were dissected as described by the Eaton-Peabody Laboratories [87]. The tissue was permeabilized with PBS-0.3% Triton X-100 (Sigma-Aldrich, St. Louis, MO, USA) and blocked in permeabilization buffer supplemented with 5% normal goat serum (Cell Signaling Technology, Danvers, MA, USA). Pre-synaptic ribbons and post-synaptic densities were labelled using a monoclonal mouse anti-CtBP2 antibody (1:200, BD Biosciences, San Jose, CA, USA) and a monoclonal mouse anti-GluR2 antibody (1:2000, Sigma-Aldrich, St. Louis, MO, USA), respectively, followed by incubation with the corresponding secondary antibodies, goat anti-mouse IgG2 Alexa Fluor^®^ 488 and goat anti-mouse IgG1 Alexa Fluor^®^ 568 (1:1000, ThermoFisher Scientific, Waltham, MA, USA). Nuclei were counterstained with DAPI. The labelled tissue was mounted with the ProLong Gold antifade reagent (ThermoFisher Scientific, Waltham, MA, USA).

### 4.10. Cochlear Frequency Mapping

Frequency mapping was performed as previously described [16]. Tissue was imaged using a Nikon Eclipse E600 fluorescence microscope (Nikon, Tokyo, Japan) equipped with an Infinity 3 camera (Lumenera, Ottawa, ON, Canada) and processing the images with the Measure Line plugin for ImageJ (version 1.52A, National Institutes of Health, Bethesda, MD, USA) developed by the Eaton-Peabody Laboratories, as described in [87] (https://www.masseyeandear.org/research/otolaryngology/eaton-peabody-laboratories/histology-core, accessed on 29 September 2020).

#### 4.10.1. Synapse Counts

Stained tissues were imaged at the regions around 8 kHz, 16 kHz, 24 kHz, 32 kHz, and 45 kHz using a Nikon W1 spinning disk confocal on a Nikon Ti2 inverted microscope (Nikon, Japan) using a 60× oil objective and 0.2 μm sections. Paired synapses were identified via the co-localization of GLUR2 and CTBP2. Synapses were counted on Z-stacks using the ImageJ Cell Counter plugin.

#### 4.10.2. Cytocochleograms

Outer hair cell nuclei counterstained with DAPI were imaged throughout the length of the cochlea using a NikonEclipse E600 microscope (Nikon, Japan) equipped with an Infinity 3 camera (Lumenera, Ottawa, Canada). The counts were expressed as percentage of missing hair cells in 2 kHz intervals and binned within the following frequency intervals: 0.0–4.0 kHz, 4.0–5.6 kHz, 5.6–8.0 kHz, 8.0–11.3 kHz, 11.3–16.0 kHz, 16.0–22.6 kHz, 22.6–32.0 kHz, 32.0–45.2 kHz, 45.2–55.0 kHz.

### 4.11. In Situ Hybridization

Tissue for the fluorescent in situ hybridization assays was obtained from intact male and female (*n* = 5 per sex) wildtype B6CBAF1/J mice (Jackson Laboratory, ME). At 10-weeks of age, mice were euthanized via CO_2_ asphyxiation followed by decapitation. Immediately following euthanasia, temporal bones were harvested from the head. The round and oval windows of the cochlea were opened, and a small opening was made in the apex cochlea to allow for increased perfusion during fixation. The temporal bones were fixed overnight at 4 °C in freshly made RNase free 4% paraformaldehyde (Alfa Aesar, Haverhill, MA, USA) in sterile 1× phosphate-buffered saline (Corning Inc., Corning NY, USA), then decalcified in 150 mM EDTA (Quality Biological, Gaithersburg, MD, USA) at 4 °C for 3 days. Decalcified ears were processed by a sucrose gradient and embedded in SCEM tissue embedding medium (Section-Lab Co., Ltd., Hiroshima, Japan). Frozen tissue was cryosectioned at 12 µm thickness on a Leica CM 1850 cryostat microtome (Leica, Vienna, Austria).

The fluorescent in situ hybridization assay was performed as described in the RNAscope™ Multiplex Fluorescent v2 protocol (Advanced Cell Diagnostics, Newark, CA, USA), using the RNAscope™ Multiplex Fluorescent Reagent Kit v2 (Cat No. 323100) and three RNAscope™ probes, ESR1 (Cat No. 478201-C3), ESR2 (Cat No. 316121-C2), and LYPD1 (Cat No. 318361). Images were taken at 40× magnification at the apical, middle, and basal turns of the cochlea using a Nikon W1 spinning disk confocal microscope (University of Maryland School of Medicine Center for Innovative Biomedical Resources [Confocal Microscopy Facility]–Baltimore, MD, USA). Image processing was performed with the Fiji ImageJ software to enhance signal clarity. 

Quantification of *Esr2* and *Lypd1* co-expression was performed using the QuPath software according to the methods described in the QuPath RNA ISH Analysis guide (Advanced Cell Diagnostics, CA). Briefly, the spiral ganglion area was circled, and individual neurons were outlined using the ‘Cell detection’ program. Puncta for *Esr2* were identified using the ‘Subcellular detection’ feature and the detection parameters were adjusted to distinguish positive signals from the background. Co-expression was defined by the number of *Esr2* puncta overlapping with *Lypd1* puncta.

### 4.12. Statistics

All statistical analyses were performed using Prism 8 software (GraphPad, San Diego, CA, USA). Analyses of auditory thresholds were completed within groups using a repeated measure (RM) 2-way ANOVA (treatment × frequency) followed by Sidak’s multiple comparison test, or between groups using a 2-way (treatment × frequency) or 3-way (treatment × frequency × biological sex) ANOVA followed by Tukey’s post-hoc test. Within-group analyses of DPOAE thresholds were completed using a 2-way ANOVA (treatment × frequency) followed by Sidak’s multiple comparison test. Analyses of wave-1 amplitudes were completed within groups at each frequency using a RM2-way ANOVA (treatment × stimulus intensity) followed by Sidak’s multiple comparison test, or between groups at each frequency using a 3-way ANOVA (treatment × stimulus intensity × noise exposure) followed by Tukey’s post hoc test. Analyses of OHC loss and paired synapses counts between groups were completed using a 2-way ANOVA (treatment × frequency) followed by Sidak’s multiple comparison test. Analyses of endocochlear potentials and RNAscope™ assays were completed using a student’s *t*-test. An adjusted *p*-value of <0.05 was set as the threshold for statistical significance for all pairwise comparisons. F-scores for main effects of treatment and biological sex are provided in Table A1.

## 5. Conclusions

In conclusion, our data suggest that gonadal E_2_ protects against NIHL, and that this E_2_-conferred protection may be sex-dependent. E_2_-replacement in gonadectomized adult female mice reduces hearing loss following both a PTS-inducing and TTS-inducing noise exposure. Histological analysis of cochlear tissue demonstrates that gonadectomy increases susceptibility to, and E_2_-replacement protects against, loss of OHC and the synaptic uncoupling of IHCs and SGNs following noise exposure. Furthermore, E_2_-replacement in gonadectomized female mice modulates the ABR and OHC physiology in the absence of noise exposure, providing additional evidence of E_2_’s multi-modal modulatory capacity. Taken together, our data present E_2_ signaling pathways as potential therapeutic targets to mitigate noise-induced hearing loss. 

## Appendix A

**Table A1:** F-scores for main effects of treatment and biological sex for the 2-way and 3-way ANOVAs.

		Main Effect	F-Score	*p*-Value
Baseline Physiology				
ABR Thresholds		Treatment	28.53	<0.0001
Wave-1 Amplitudes				
	8 kHz	Treatment	57.07	<0.0001
	16 kHz	Treatment	39.34	<0.0001
	24 kHz	Treatment	43.79	<0.0001
	32 kHz	Treatment	32.35	<0.0001
DPOAE Thresholds				
	Vehicle Condition	Treatment	0.1903	0.6671
	E2 Condition	Treatment	36.11	<0.0001
Paired Synapses		Treatment	0.7923	0.3903
PTS Experiments				
ABR Thresholds				
	Baseline	Sex	0.1007	0.7516
		Treatment	0.005573	0.9406
	CTS	Sex	21.04	<0.0001
		Treatment	17.15	<0.0001
	PTS	Sex	27.25	<0.0001
		Treatment	33.55	<0.0001
OHC Loss		Treatment	9.791	0.0019
Paired Synapses		Treatment	3.009	0.1033
TTS Experiments				
ABR Thresholds				
	Baseline	Treatment	2.144	0.1465
	CTS	Treatment	34.03	<0.0001
	TTS	Treatment	0.09137	0.7631
Wave-1 Amplitudes				
	8 kHz	Treatment	30.99	<0.0001
	16 kHz	Treatment	42.32	<0.0001
	24 kHz	Treatment	59.31	<0.0001
	32 kHz	Treatment	58.97	<0.0001
OHC Loss		Treatment	5.517	0.0278
Paired Synapses		Treatment	6.705	0.0100
